# Development and validation of a self-regulation scale within the German version of the Early Development Instrument

**DOI:** 10.1186/s12887-023-04334-1

**Published:** 2023-10-16

**Authors:** Sabine Georg, Bernd Genser, Joachim Fischer, Steffi Sachse, Freia De Bock

**Affiliations:** 1 Medical Faculty Mannheim, Center for Preventive Medicine and Digital Health (CPD), Division of General Medicine, Old Brewery, Heidelberg University, Röntgenstraße 7, 68167 Mannheim, Germany; 2 Department of Developmental Psychology, Heidelberg University of Education, Keplerstraße 87, 69120 Heidelberg, Germany; 3 Department of General Pediatrics, Neonatology and Pediatric Cardiology, Medical Faculty, University Hospital Düsseldorf, Heinrich-Heine-University, Düsseldorf, Germany; 4 BGStats Consulting, Schleifmühlgasse 7, 1040 Vienna, Austria

**Keywords:** Self-regulation, Child preschool [MeSH, Child development [MeSH, Early Development Instrument, Germany, Environment and public health [MeSH, Monitoring

## Abstract

**Background:**

Early childhood self-regulation (SR) is key for many health- and education-related outcomes across the life span. Kindergarten age is a crucial period for SR development, and within this developmental window, potential SR difficulties can still be compensated for (e.g., through interventions). However, efficient measurement of SR through brief, comprehensive, and easy-to-use instruments that identify SR difficulties are scarce. To address this need, we used items of an internationally applied kindergarten teacher questionnaire—the Early Development Instrument (EDI) – to develop and validate a specific SR measurement scale.

**Methods:**

The psychometric evaluation and validation of the selected SR-items was performed in data collected with the German version of the EDI (GEDI), in two independent data sets – (a) the development dataset, with 191 children, and b) the validation dataset, with 184 children. Both included three- to six-year-old children and contained retest and interrater reliability data. First, three independent raters—based on theory—selected items eligible to form a SR scale from the two SR-relevant GEDI domains "social competence" and "emotional maturity". Second, exploratory and confirmatory factor analysis using structural equation modeling examined the item structure across both data sets. This resulted in a defined SR scale, of which internal consistency, test–retest and interrater reliability, cross-validation, and concurrent validity using correlation and descriptive agreements (Bland–Altman (BA) plots) with an existing validated SR-measuring instrument (the *Kindergarten Behavioral Scales*) were assessed.

**Results:**

Confirmatory factor analysis across both data sets yielded the best fit indices with 13 of the GEDI 20 items initially deemed eligible for SR measurement, and a three-factor structure: a) behavioral response inhibition, b) cognitive inhibition, c) selective or focused attention (RMSEA: 0.019, CFI: 0.998). Psychometric evaluation of the resulting 13-item-GEDI-SR scale revealed good internal consistency (0.92), test–retest and interrater reliability (0.85 and 0.71, respectively), validity testing yielded stability across populations and good concurrent validity with the *Kindergarten Behavioral Scales* (Pearson correlation coefficient: mean 0.72, range 0.61 to 0.84).

**Conclusions:**

The GEDI contains 13 items suitable to assess SR, either as part of regular EDI developmental monitoring or as a valid stand-alone scale. This short 13-item (G)EDI-SR scale may allow early detection of children with SR difficulties in the kindergarten setting in future and could be the basis for public health intervention planning. To attain this goal, future research should establish appropriate reference values using a representative standardization sample.

## Introduction

Self regulation (SR) is a fundamental developmental skill impacting a child’s performance and health across the lifespan [1, 2]. *It describes the ability to adapt one's thoughts, feelings, and behavior to the demands of a particular situation in order to optimally pursue personal goals* [3]. Moreover, *SR refers to processes that enable us to maintain optimal levels of emotional, motivational, and cognitive arousal. It […] overlaps substantially with inhibitory control*, a core dimension of executive functions [4].

From a medical, psychological and pedagogical perspective, good SR skills are considered a protective factor regarding mental [5–7] and physical health [8] and have been found to longitudinally predict health, success in professional and private life, satisfaction with life and social equity in adulthood [1].

Accumulating evidence in the last two decades suggests that more and more children from school age to adolescence have difficulties in regulating their behaviors [9]. For example, the prevalence of behavioral and psychological problems related to SR in kindergarten and primary school has been steadily increasing [2, 10–12]. This not only presents challenges for the daily work of teachers [13–15], but studies also suggest that these problems persist into adolescence with a 50% chance [16], resulting in a high societal burden and possible medical costs [17, 18].

With the window for promoting children’s SR skills opening years before entering school, early identification of children with SR difficulties combined with early intervention e.g. in kindergarten seems key from a public health perspective. As SR development depends on environmental factors and experiences [19–21] (besides biological maturity), interventions that change the environment and experiences have the potential to effectively support child SR development [22–24]. Current systematic reviews have shown effectiveness of different SR promoting interventions in early childhood education and care environments (ECECs) [23, 24]. Other studies showed that supportive environmental factors such as high-quality teacher–child interaction [25] are positively associated with SR development in children. This suggests that a public health approach combining the efficient identification of children with SR difficulties early on with the implementation of effective interventions in the kindergarten setting has a high potential.

To identify vulnerable children, valid measurement of SR in kindergartens is necessary. As SR skills are part of psychological and social-emotional child development, questionnaires that are used to assess the latter might be promising. These include the Behavioral and Emotional Rating Scale (BERS, 26 items, domains: behavioral self-control, emotional self-control) [26], the Child Behavior Checklist (CBCL, 33 items, domains: emotionally reactive, attention problems, aggressive behavior) [27], the Child Behavior Questionnaire (CBQ, 12 items, domains: attentional focusing, inhibitory control) [28], the Child Behavior Rating Scale (CBRS, 17 items, domains: self-regulation, social/interpersonal skills) [29], Conners' rating scale – teacher form (CTRS, 28 items, domains: conduct problems, day-dreaming inattention, anxious fearful, hyperactivity) [30], the Devereux Early Childhood Assessment (DECA, 8 items, domain: self-control) [31], Social competence and behavior evaluation—preschool edition (SCBE, 20 items, domains: anger-aggression, social competence) [32], the Social Competence Scale (SCS, 13 items, domains: prosocial behavior, emotion regulation) [33], the Strengths and difficulties questionnaire (SDQ, 25 items, domains: emotional symptoms, conduct problems, hyperactivity/inattention, peer relationship problems, prosocial behavior) [34, 35], and the Behavior Rating Inventory of Executive Function—Preschool Version (BRIEF-P, 63 items, domains: inhibition, attention shift, emotional control, working memory, planning/organizing) [36]. Although many instruments might be available to measure SR skills, the most important ones were suggested to be the CBQ, BRIEF, CBCL and SDQ [37]. However, from a public health perspective, all of these are too comprehensive and long (e.g. number of items for SR measurement = 12, 26 23, 25, respectively) for screening purposes, and do not feature SR as a separate construct.

Several of these questionnaires also exist in German, e.g. the SDQ or the BRIEF-P [38]. Furthermore, additional questionnaires exist that were developed in the German context and are primarily used in Germany, such as the Kindergarten Behavior Scales (VSK, 49 items, domains: anxiety, hyperactivity and inattention, aggressive behavior, emotional dysregulation, social competence, emotional knowledge/empathy, self-regulation) [39], the Organizing Education in Kindergarten screening (BIKO, 33 items six domains: willingness to cooperate with educational staff, integration into the group, problem behavior towards peers, prosocial behavior towards peers, play and task behavior, regulation of emotions) [40, 41], the Dortmund Developmental Screening for Kindergarten (DESK 3–6 R, 45 to 50 items depending on age, domains: fine motor skills, gross motor skills, social competence, social behavior, social interaction, attention and concentration, cognition and language, cognition, basic competence literacy, basic competence numeracy, language and communication) [41] or the questionnaire Competencies and Interests of Children (KOMPIK, 158 items across 11 domains: motor skills, social and emotional behavior, motivation, language and early literacy, maths, science, music, design, health, well-being, and social relationships) [42].

While these instruments meet scientific standards, they are all longer and quite time-consuming (minimum 40 items, while the DESK even contains performance tasks *over and above* questionnaire items, which requires even more time and a suitable physical environment in kindergartens). In addition, most of them do not feature SR as a separate construct and are far too comprehensive (e.g. measure development or behavioral issues in general), which reduces their suitability as efficient SR screening tools in the kindergarten environment and also might explain why they failed to gain wide use in Germany.

To move the field of developmental monitoring and public health intervention planning in kindergartens in Germany forward, we previously adapted the internationally widely used Canadian Early Development Instrument (EDI) [43] to the German context and published the German version of the EDI (GEDI) [44]. The EDI is a valid and reliable teacher 103-item questionnaire assessing a child’s ability to meet age-appropriate development expectations in five domains (see below), developed by Magdalena Janus and colleagues at the Offord Center for Child Studies at McMaster University, Ontario. The instrument was designed as a screening and developmental monitoring tool [45–49]. It serves to collect data on the development of 3- to 6-year-old children in all relevant developmental domains [50]. In Canada and other countries, the EDI is integrated into a public health monitoring and intervention planning approach, which results in a tailored implementation of interventions in kindergartens to support child health and development.

Based on the features described above, the EDI could provide an optimal basis to develop a brief, but psychometrically sound and fully questionnaire-based screening instrument to detect SR difficulties in kindergarten children. In addition, the worldwide use of the EDI would allow to assess SR as part of the regular EDI monitoring in kindergartens in many countries.

Therefore, this study assesses whether it is possible to develop a valid scale measuring SR by recombining items of the theoretically relevant EDI domains "social competence" and "emotional maturity". The following research questions guide our study:

- Can existing items from the (G)EDI be selected based on solid theoretical and conceptual considerations and recombined to form a valid (stand-alone) SR scale?

b) Does the resulting (G)EDI-SR scale have adequate psychometric properties and validity?

## Methods

### Recruitment, data collection and sample description

The present study collected data with the (G)EDI teacher questionnaire [43, 44] in two independent data sets – (a) the development dataset, with 191 children, collected in June 2016 to pilot the EDI in Germany in three different towns, with more details on recruitment and psychometric features published elsewhere [44], and b) the validation dataset, with 184 children, collected in fall 2021, in kindergartens in a small town in the South-West of Germany (population approx. 15.000), which intended to use the GEDI as the starting point for a community-based early childhood prevention strategy. In both data collections, teachers completed the full GEDI and the VSK-SR subscale for all participating children. The precondition to fill out the GEDI was that the teachers knew the children for at least one month, had sufficient command of the German language, and took part in a training session prior to the assessment. The previous training ensured that all teachers had the same level of knowledge about the instrument, its purpose and completion.

All data were collected electronically and given an individual pseudonym by the teachers to match first and second surveys to the same child with a 100% degree of accuracy.

Eligibility criteria for the children to whom the GEDI was administered comprised age 3 to 6 years, the presence of written informed parental consent and the absence of special needs. Table 1 displays descriptive characteristics for both samples and provides the number of eligible and finally participating children and teachers. Ethical approvals for both data collections were granted by the Ethics committee of the Medical Faculty Mannheim, Heidelberg University (development sample: 2015-640N-MA; validation sample: 2016-588N-MA). The teachers’ participation was taken as an implicit consent to participate in our study.

**Table 1:** Characteristics of development and validation samples

		**N development sample (%)**	**N validation sample (%)**
Eligible (invited)	Children	444	385
	Kindergartens	9	6
	Teachers	60	75
Participating	Children with parental consent	225 (51)	209 (54)
	Kindergartens	9 (100)	6 (100)
	Teachers	60 (100)	33(44)
	Cases excluded upon reasons	34^a^ (15)	25^b^ (12)
	Cases in dataset	191 (43)	184 (48)
	mean age (range; SD)	4.27 (3 to 6; 1.05)	4.25 (3 to 6; 0.94)
	n 3 years	58 (30)	46 (25)
	n 4 years	60 (31)	65 (35)
	n 5 years	43 (23)	55 (30)
	n 6 years	30 (16)	18 (10)
	Gender (female)	49%	51%
	German second language	18%	7%
	SES low/middle/high	2,6/49,2/40,3%	-

^a^*n* = 5 with missing data or a “don’t know” response to the special needs assignation variable; *n* = 28 with special needs assignation, *n* = 1 under the age of three

^b^*n* = 22 due to an affirmative answer to the special needs question, *n* = 3 under the age of three

SES = socioeconomic status

### Study design – overview

In a first step, the selection of GEDI items that theoretically map to SR was performed, which resulted in eligible GEDI-SR items. To assess the construct and dimensions of the eligible GEDI-SR items (see beneath), we used the development dataset, resulting in a first GEDI-SR scale. The GEDI data from the two independent samples were then used to cross-validate the item and factor structure of the GEDI-SR scale from the development data set to the validation data set. In a next step, using the validation data set, the GEDI-SR scale was compared with the VSK-SR items to assess concurrent validity of the GEDI-SR scale. Moreover, our reliability analyses used data from repeated retests of the GEDI within the validation sample. In the following, measurements and related statistical analyses for the different steps of the study design are presented in more detail.

### Measurements

#### The GEDI as basis for SR scale development

The GEDI, like the original EDI, is a kindergarten teacher questionnaire to assess early childhood development in the following domains: “physical health and well-being" (13 items), "social competence" (26 items), "emotional maturity" (30 items), "language and cognitive development" (25 items), and "communication and general knowledge" (8 items) based on accumulated teacher impression and observation (and *not* on performance tasks). As a public health tool, the (G)EDI can be helpful in several ways: e.g. for teachers to create optimal learning opportunities tailored to individual child developmental profiles, for school boards and ministries to plan resource allocations to kindergartens (e.g. child-teacher relation) and to describe specific intervention needs in kindergartens which could be used for public health monitoring and planning (including to convince funders of intervention projects) [51].

The validation of the GEDI in the German context across the original five main domains demonstrated excellent internal consistency (0.73 < α > 0.99), moderate to good test–retest and interrater reliability (0.50 to 0.81 and 0.48 to 0.71, respectively [*p*-value < 0.05]), and good concurrent validity with other developmental instruments (range: 0.32 to 0.67) (details see [44]).

However, focus groups with teachers after the first data collection in Germany revealed a need to provide age-specific ratings (the original instrument is applied to 5-year old children in their preschool year, while in Germany kindergartens serve children from the age of 3 to 6). Using item response analyses, appropriateness of age-related information content and redundancies (e.g. some items from the original 103 items that did not provide additional content for specific age groups) were resolved, which thereby led to an overall shortening of the GEDI as compared to the EDI. The age-adjusted, age-specific and shorter GEDI contains different numbers of items, depending on the age group: *n* = 69 for 3–4 year-olds, *n* = 65 for 5-year-olds, and *n* = 61 for 6-year-olds. In the present study, only the items of the SR-relevant domains of the GEDI, "social competence" (*n* = 15 and 16 items for 3–4- as well as 5–6-year-olds, respectively) and "emotional maturity" (*n* = 21 items for all age groups), were considered and analysed.

#### The VSK as measure to assess concurrent validity

Besides the GEDI we applied the SR subscale of the German *Kindergarten Behavioral Scales* (Verhaltensskala für den Kindergarten = VSK-SR) [39] to assess concurrent validity. The VSK comprises 49 items in seven domains: anxiety, hyperactivity and inattention, aggressive behavior, emotional dysregulation, social competence, emotional knowledge/empathy, self-regulation). The VSK-SR scale entails five items, with an internal consistency of = 0.79: *waits for his or her turn, performs activities he or she does not like, wants things immediately, considers the consequences of his or her own actions, finishes tasks*. The concurrent validity of the VSK-SR subscale was assessed with the SDQ [35] and proved to be moderate (-0.67, *p*-value < 0.001) and thus acceptable [52].

#### Selection of items: Assessing eligibility and selecting SR-mapping GEDI items

We used a theory-based approach to identify items that might be relevant for the development of a SR scale. As a theoretical basis, we used a widely accepted categorization system of SR [4]. It considers SR as a multidimensional latent construct, including three closely related sub-dimensions: a) cognitive inhibition, which means the inhibition of thoughts and memories, b) selective or focused attention, or c) response inhibition: self-control/discipline. With these definitions in mind, three independent raters who were professionally familiar with early childhood development (childhood education, occupational therapy, developmental psychology) assessed all items within the GEDI domains of "social competence" and "emotional maturity”, which deemed relevant as these skills are closely related to SR skills [53]. Each item was labeled each as either 0 (not mapping to SR) or 1 (mapping to SR). Subsequently, they assigned the items mapping to SR to the three sub-dimensions of SR. Interrater agreement was assessed using kappa-statistics. Inconsistencies were resolved through discussion including a third independent rater until consensus was reached. This process resulted in items eligible to form the new GEDI-SR scale.

#### Statistical analyses

##### Operationalization and categorization of responses in the GEDI-SR scale

Like in the original EDI, we retained three-point Likert scales for the GEDI (coding: often/very true = 10, sometimes/somewhat true = 5, and never/not true = 0) [43]. Higher mean scores indicated better development. Children were excluded from analyses in a domain if ≥ 30% of values were missing [20]. In the absence of a normative German sample to establish valid cut-offs, and in line with the original EDI procedures, children who scored lower than the 10th percentile in the ensuing GEDI-SR scale were preliminarily deemed as “vulnerable” in terms of SR [54].

##### Descriptive analysis of the two data sets

We initially compared descriptive statistics of the development and validation datasets (sample size, mean age, distribution and scorings at 10^th^, 25ths, 50^th^ and 75^th^ percentile) using kernel density plots to reveal differences that might further help to explain potential inconsistencies in structured equation modeling (SEM).

##### Assessment of construct and dimensions of the eligible GEDI-SR items: Psychometric evaluation

We first performed an exploratory and confirmatory factor analysis. Using the development dataset, we applied the measure of sampling adequacy (MSA, < 0.5 unsuitable, ≥ 0.6 usable, > 0.8 good [55]). To test the hypothesis regarding the factor structure among the eligible GEDI-SR items, we conducted an exploratory factor analysis using structural equation modeling (maximum-likelihood method). The comparative fit index (CFI, > 0.95) [56] and the root mean squared error of approximation (RMSEA, < 0.05) [57–60] served as goodness-of-fit indicators of the model. To avoid overfitting, we tested the model fitted with the development dataset by recalculating the same model using the validation dataset. We aimed to replicate the main structured equation modeling composition of the model (confirmatory factor analysis). Since we were still in the exploration stage, we adjusted correlations among items in the validation dataset where necessary in favor of a better model fit.

##### Reliability testing of the GEDI-SR scale

We assessed internal consistency (Cronbach’s alpha) of the GEDI-SR scale resulting from the confirmatory factor analysis and used intraclass correlation coefficients (ICC) to assess test–retest and interrater reliability (0.5 = poor, 0.5 to 0.75 = moderate, 0.75 to 0.9 = good, and > 0.9 = excellent [61]. We asked teachers to repeat the GEDI for a randomly selected subset of children (*n* = 72; 3 children per age group) after two weeks. ICCs indicate the strength of the correlation of the GEDI-SR scores between the two measurement time points. The higher the ICC value, the better the correlation between T1 and T2 and the better the corresponding reliability. Additional plausibility checks using invariant demographic variables (birth quarter, gender) ensured the accuracy between T1 and T2 data.

##### Concurrent validity testing of the GEDI-SR scale

We assessed concurrent validity by means of Pearson correlation coefficients and plotting differences between the mean GEDI-SR and VSK-SR scores using Bland–Altman (BA) plots for each age group. BA plots are graphical representations that can be used to compare two measurement methods by analyzing the agreement between these: a difference plot combined with calculation of the two (upper and lower) limits of the differences between the methods (the so-called 95% limits of agreement). The x axis shows the mean of the results of the two methods ([A + B]/2), whereas the y axis represents the absolute difference between the two methods ([B—A]) [62, 63]. The closer the points in the plot are aligned around the line of mean difference (line centered at zero of the y-axis), the better the agreement. A good agreement is to be interpreted as good concurrent validity.

To meet the requirement for normality [64, 65], we used the Stata commands *gladder* and *qladder* and selected the closest to normal distribution. To enable cross-measure comparisons in BA plots, GEDI-SR and VSK-SR scores were transformed into z-scores. BA plots were generated using the Stata command *concord* [66]. The association between the two measures was examined by (i) considering the mean difference and (ii) the scattering of dots around the mean difference line in relation to the latent trait continuum on the x-axis.

All analyses were conducted using Stata (StataCorp. 2015. Stata Statistical Software: Release 15. College Station, TX: StataCorp LP.).

## Results

### Results of the item selection process

The theory-based item selection resulted in a list of 20 eligible GEDI-SR items (Table 2). In the selection process, a moderate kappa of 0.5 between the three raters could be achieved.

**Table 2:** GEDI items to develop a self-regulation scale selected on a theoretical basis

**Original GEDI-Domain**		**Items** ***Would you say that this child…***
Social competence	qc2	Has the ability to get along with peers
	qc5	Follows rules and instructions
	qc7	Demonstrates self-control
	qc9	Demonstrates respect for adults
	qc10	Demonstrates respect for children
	qc11	Accepts responsibility for actions
	qc12	Listens attentively
	qc14	Completes work on time
	qc15	Works independently
	qc16	Takes care of school materials
	qc17	Works neatly and carefully
	qc24	Is able to follow class routines without reminders
Emotional maturity	qc37	Gets into physical fights
	qc42	Can’t sit still, is restless
	qc43	Is distractible, has trouble sticking to any activity
	qc44	Fidgets
	qc46	Has temper tantrums
	qc47	Is impulsive, acts without thinking
	qc48	Has difficulty awaiting turn in games or groups
	qc50	Is inattentive

### Assessment of construct and dimensions of the eligible GEDI-SR items: Psychometric evaluation

The measure of sampling adequacy analysis amounted to MSA = 0.9. Exploratory factor analysis with the development sample revealed three highly significant (*p*-value < 0.001) interrelated factors (Table 3). The explanations in the right column of this table show that the loadings and allocations of the eligible items to the factors are theory-based and comprehensible. The contents of all items with loadings higher than or equal to 0.4 could be transparently assigned to the corresponding factors. Four items with loadings below 0.4 had too general a wording and their content did not necessarily refer to the ability to self-regulate. Therefore, they were removed from consideration leaving us with 16 of the initially 20 eligible items. Based on the theoretical background, the ensuing three factors were labeled as: 1) behavioral response inhibition; 2) cognitive inhibition; 3) selective or focused attention.

**Table 3:** Factor loadings and theory-based explanations resulting from exploratory factor analysis with the development dataset

Variable	Would you say that this child…	Factor1	Factor2	Factor3	Uniqueness	Theory based explanation
qc10	Demonstrates respect for children	0.7440			0.4707	Requires to inhibit emotions and behavior
qc9	Demonstrates respect for adults	0.6693			0.4933	Requires to inhibit emotions and behavior
qc37	Gets into physical fights	0.6625			0.6026	Requires to regulate emotions and needs a certain motivation to regulate behavior
**qc47**	Is impulsive, acts without thinking	0.6237			0.5022	Impulsivity is the inability to regulate emotions and behavior. If someone is planned, then he can regulate his emotions and act in a self-controlled manner
**qc5**	Follows rules and instructions	0.5344			0.6048	Requires the ability to motivate oneself to adapt and to inhibit "rebellious" emotions and behave accordingly
**qc11**	Accepts responsibility for actions	0.4134			0.4878	Requires the ability stand up for own mistakes to resist the impulse to be offended and "run away". This requires to regulate emotions and behavior by being honest and not offended
qc7^a^	Demonstrates self-control	< 0.4			0.6445	Can mean anything and does not separate well. The item is not worded accurately enough
qc46^a^	Has temper tantrums	< 0.4			0.8090	You can throw tantrums for very different reasons. However, this does not necessarily mean that one has a bad SR
qc15	Works independently		0.7598		0.4631	to be able to work independently, I have to be able to remember things and stay on task
qc17	Works neatly and carefully		0.7359		0.3816	to be neat and careful, I need to be able to structure myself and my thoughts
**qc14**	Completes work on time		0.7344		0.4662	To stay on schedule, I also need to be able to stay on task and focus my thoughts on what I'm doing
qc24	Is able to follow class routines without reminders		0.5504		0.6140	Requires the ability to remember things and also be able to recall it again
qc12	Listens attentively		0.5315		0.4751	Requires the ability to block out disturbing thoughts and memories
qc16	Takes care of school materials		0.5002		0.4667	Requires to be careful and not destroy anything on purpose. Requires the ability to suppress the impulse to destroy, which is sometimes perceptible, and behave appropriately and in a controlled manner
qc2^a^	Has the ability to get along with peers		< 0.4		0.7658	Too many things in one item. Doesn't have to be SR ability if someone can get along with another kid
qc44	Fidgets			0.7379	0.4575	Fidgeting and being restless and physically active doesn't necessarily mean that a child is not able to concentrate, but certainly often goes hand in hand with it
**qc43**	Is distractible, has trouble sticking to any activity			0.7372	0.4048	Requires the ability to concentrate and focus attention
qc42	Can’t sit still, is restless			0.7171	0.4387	These children have difficulties to focus their attention
qc50	Is inattentive			0.6705	0.4803	These children can't concentrate and selectively focus their attention
**qc48**^a^	Has difficulty awaiting turn in games or groups			< 0.4	0.6657	Awaiting turn requires patience and waiting is different from attention and concentration

Note: ^a^Item excluded from subsequent analysis (structured equation modeling and BA-Plots)

Item numbers in bold: Items corresponding with VSK-SR items: *Waits for his or her turn (qc5), Performs activities he or she does not like (qc11), Wants things immediately (qc48), Considers the consequences of his or her own actions (qc11, qc47), Finishes tasks (qc14, qc43)*

Confirmatory factor analysis with the development dataset using structured equation modeling revealed highly significant correlations at the factor and item level. Three items loaded below 0.6 and were therefore excluded from the final model (Table 4) leaving us with 13 items of the initially 20 eligible items. The good model fit (RMSEA: 0.029, CFI: 0.993) is presented in Table 4, resulting in a 13-item SR scale to be tested further.

**Table 4:** Factor structure, Item- and Subdomain correlations of the latent construct self-regulation using structured equation modeling

			**development dataset**						**replication with validation dataset**					
			*N* = *191*						*N* = *184*					
Factor	Item		Coefficient (subdomain level)	Coefficient (item level)	SE	95% CI lb	95% CI ub	Correlations with other items	Coefficient (subdomain level)	Coefficient (item-level)	SE	95% CI lb	95% CI ub	Correlations with other items
sd16 discipl	qc10	Demonstrates respect for children	sd17 0.76*** sd18 0.62***	0.64***	0.05	0.54	0.75	qc9 0.26** qc15 -0.14 (ns) qc14 -0.19* qc16 0.13 (ns)	sd17 0.66*** sd18 0.77***	0.56***	0.06	0.45	0.68	qc9 0.52*** qc16 0.39***
	qc9	Demonstrates respect for adults		0.67***	0.05	0.56	0.77			0.49***	0.06	0.37	0.61	qc16 0.29*** qc42 0.19*** qc50 -0.15*
	qc37	Gets into physical fights			< 0.6	excluded								
	**qc47**	Is impulsive, acts without thinking			< 0.6	excluded								
	qc5	Follows rules and instructions		0.68***	0.05	0.58	0.78	qc15 -0.19*		0.97***	0.06	0.86	1.08	qc11 -1.67(ns) qc16 0.58(ns)
	q11	Accepts responsibility for actions		0.72***	0.05	0.63	0.81			0.86***	0.06	0.74	0.98	qc15 0.43*** qc17 0.38** qc16 0.63***
sd17 cog	qc15	Works independently	sd18 0.57***	0.5	0.05	0.49	0.71	qc17 0.13 (ns) qc14 0.225** qc44 -0.15*	sd18 0.89***	0.69***	0.05	0.59	0.79	qc17 0.23** qc14 0.13(ns) qc12 -0.28* qc42 -0.35***
	**qc17**	Works neatly and carefully		0.6***	0.04	0.76	0.90	qc12 -0.35**		0.71***	0.05	0.62	0.80	qc12 -0.49*** qc42 -0.18* qc50 0.14(ns)
	**qc14**	Completes work on time		0.68***	0.05	0.59	0.77	qc16 -0.19*		0.69***	0.04	0.61	0.77	
	qc24	Is able to follow class routines without reminders			< 0.6	excluded								
	qc12	Listens attentively		0.74***	0.04	0.65	0.82	qc43 0.28**		0.86***	0.03	0.81	0.92	
	qc16	Takes care of school materials		0.76***	0.04	0.68	0.83			0.67***	0.04	0.58	0.75	qc42 -0.14*
sd18 att	qc44	Fidgets		0.64***	0.05	0.54	0.75	qc42 0.29***		0.73***	0.04	0.65	0.81	qc42 0.45***
	qc43	Is distractible, has trouble sticking to any activity		0.81***	0.04	0.73	0.89			0.87***	0.02	0.82	0.92	
	**qc42**	Can't sit still, is restless		0.71***	0.05	0.61	0.80			0.76***	0.04	0.69	0.83	
	qc50	Is inattentive		0.71***	0.05	0.62	0.80			0.77***	0.03	0.70	0.84	
RMSEA /CFI			0.029 / 0.993						0.019 / 0.998					

*sd*  Subdomain, *SE* Standard error, *CI* Confidence interval, *RMSEA* Root mean square error of approximation, *CFI* Comparative fit index

sd16 = Behavioral response inhibition

sd17 = Cognitive inhibition

sd18 = Selective or focused attention

Note: *p < 0.05, ** *p* < 0.01, ***p = 0.000

### Cross-validation: confirmatory analysis using the validation dataset

We tried to replicate the GEDI-SR scale model using the validation dataset. This cross-validation yielded similar results (RMSEA: 0.019, CFI: 0.998) (Table 4.), confirming the 13-item scale within a three-factor model structure.

### Comparison of the 13-item GEDI-SR scale’s descriptive data across the datasets

**Fig. 1:**
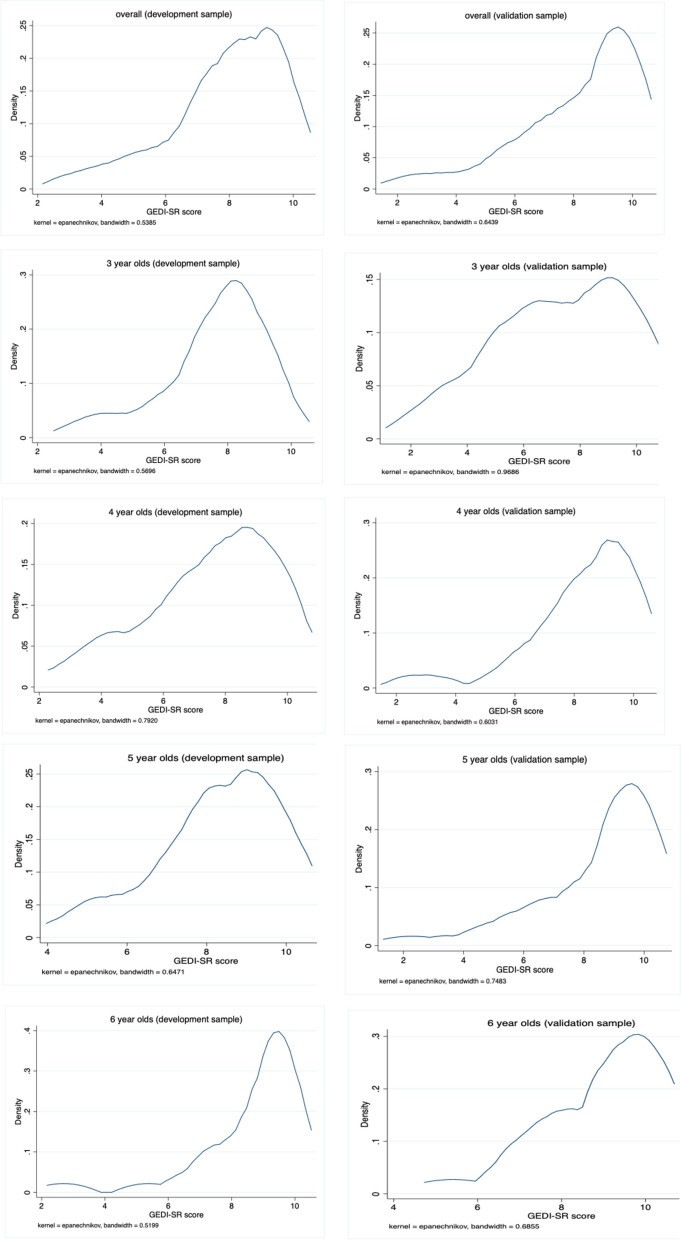
Kernel Density plots of distribution for both the development and validation datasets

Overall, descriptive statistics and age-specific kernel density plots for development and validation samples (Table 5, Fig. 1) illustrate the underlying distribution of the data. The mean value of the 10% cut-off in the samples ranged from 5.00 in the development data set to 5.42 in the validation data set, respectively. The graph shows the similarly skewed distribution in both datasets except for 3- and 4-year old children, whose percentile values partially differ from each other up to 1.4 points.

**Table 5:** Descriptive statistics for both the development and validation datasets

Participant information		GEDI-SR scale scores						
**Age**	**N**	**Mean**	**SD**	**Min**	**Max**	**10th**	**25th**	**75th**
*development sample*								
3	58	7.60	1.62	3.08	10	5.00	6.92	8.85
4	60	7.52	2.02	3.08	10	4.42	6.54	9.23
5	43	8.28	1.53	4.62	10	5.77	7.31	9.62
6	30	8.68	1.60	2.69	10	6.92	8.08	9.62
**overall**	**191**	**7.90**	**1.78**	**2.69**	**10**	**5.00**	**6.92**	**9.23**
*validation sample*								
3	46	7.26	2.31	2.08	10	3.75	5.83	9.58
4	65	8.26	1.84	2.08	10	5.83	7.50	9.58
5	55	8.44	1.98	2.08	10	5.42	7.50	10
6	18	8.96	1.36	5.42	10	7.50	7.92	10
**overall**	**184**	**8.13**	**2.03**	**2.08**	**10**	**5.42**	**7.08**	**10**

### Internal consistency, test–retest and interrater reliability results

Internal consistency (range: 0.89 < ⍺ > 0.92), overall test–retest ICC (0.85, 95%-CI: 0.71 to 0.93), and overall interrater ICC (0.71, 95%-CI: 0.43 to 0.89) of the 13-item GEDI-SR scale were good (Table 6). For test–retest and interrater reliability we obtained 27 (38%) retest pairs and 26 (36%) interrater pairs (children at least 3 years old, without special needs). The interval between T1 and T2 ranged from 6 and 9 to 30 and 22 days, respectively. Attempting to balance between "include as many pairs as possible" and "the interval between T1 and T2 should be as close to 14 days as possible" we only included pairs with a time interval between 13 and 15 days (*n* = 25 and 17 pairs). Due to a large score difference between T1 and T2 in some pairs, retest ICCs could not be calculated for 6-year-olds and interrater ICCs could only be calculated for 3-year-olds. Therefore, we only report the overall ICCs in Table 6.

**Table 6:** Reliability of the GEDI-SR scale

	**age**	**N**	**Cronbach's alpha**
Internal consistency	3 y	46	0.92
	4 y	65	0.9
	5 y	55	0.92
	6 y	18	0.89
	overall	184	0.92
		**N (pairs)**	**ICCs (CI)**
Test–retest reliability	across age groups	25	0.85 (0.71 to 0.93)
Interrater reliability		17	0.71 (0.43 to 0.89)

### Concurrent validity

**Fig. 2:**
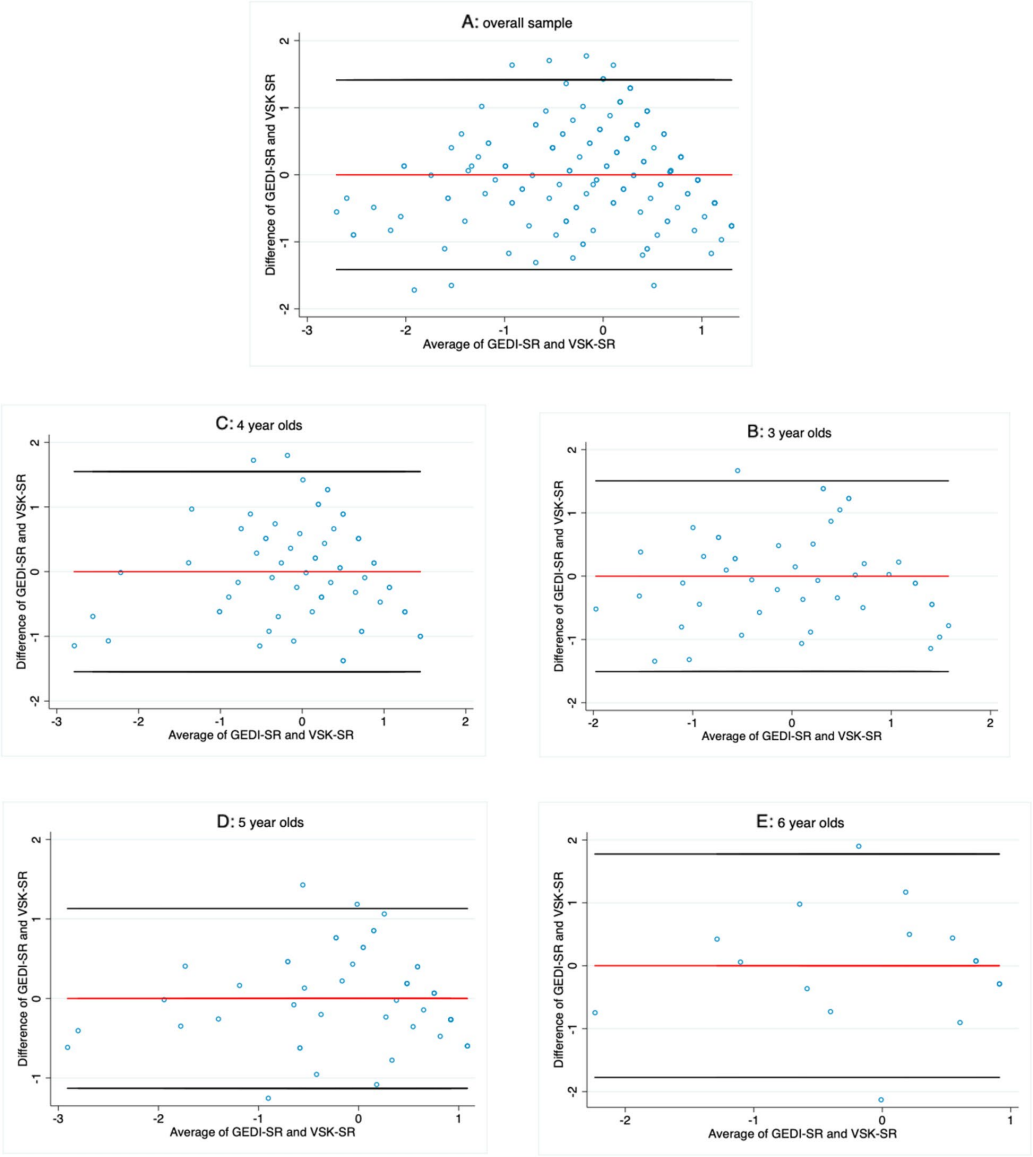
Bland–Altman plots and agreement between the GEDI-SR and VSK-SR scales’ score pairs. The metric for both x- and y-axes in each graph is the z-score for mean domain scores and the difference between scores, respectively. The line centered at zero of the y-axis marks the mean difference

Table 7 shows the results from assessing concurrent validity. With one exception, correlation coefficients indicate strong, statistically significant positive linear correlations in all age groups (range: 0.61 to 0.84). Limits of agreement are furthest apart for 6-year-olds and closest for 5-year-olds (Table 8.). Figures 2 A to E illustrate the extent to which the paired variables match. The more dispersed scatter of points around the mid-section in Figures A, B, C, and E reveal that the poorest agreement is for children with average SR skills. Children with lower average SR skills (scores <  − 1 on the x-axis) and those with higher average SR skills (scores > 1 on the x-axis) tend to be underestimated with the GEDI-SR scale compared to the VSK-SR scale. In plot D (5-year-olds), dots are clustered more tightly around the line of mean difference in the mid-section of the x-axis, indicating good agreement between the GEDI-SR and VSK-SR scales in the latent trait section, where the vast majority of children scored. For children with extreme values around -3, the plot shows a larger measurement error to the extent that the GEDI-SR scale underestimates children in the lower latent trait range.

**Table 7:** Pearson correlation between the GEDI-SR scale and the VSK-SR scale

		**GEDI-SR scale**				
		3 years	4 years	5 years	6 years	Overall
**VSK subdomain SR**	3 years	0.72***				
	4 years		0.70***			
	5 years			0.84***		
	6 years				0.61**	
	overall					0.75***

Note: *VSK* „Kindergarten Behavioral Scales “, *GEDI*  German version of the Early development instrument, *SR* Self-regulation, *** *p* = 0.000

**Table 8:** Concurrent validity: Mean differences between GEDI-SR scale and VSK-SR scale

**Age-group**	**N**	**Difference Average /** Mean difference	**SD**	**95%-Limits of agreement**	**Concordance correlation coefficient**
					Pearson's r (95%-CI)
3 years	46	-0.000^a^	0.75	-1.47 to 1.47	0.72*** (0.54 to 0.83)
4 years	65	-0.000^a^	0.77	-1.52 to 1.52	0,70*** (0.55 to 0.81)
5 years	55	0.000	0.56	-1.11 to 1.11	0,84*** (0.74 to 0.90)
6 years	18	-0.000^a^	0.92	-1.8 to 1.8	0.58** (0.17 to 0.82)
overall	184	0.000	0.71	-1.38 to 1.38	0.75*** (0.68 to 0.81)

Note: *SD* Standard deviation, *CI* Confidence interval, *** = *p* < 0.001; ^a^ values have a slightly negative tendency, which only becomes apparent after the fourth comma position

## Discussion

The aim of the study was to identify items eligible for SR-measurement within the (G)EDI domains "social competence" and "emotional maturity" by a theory-based selection process, and therefrom develop a GEDI-SR scale and assess its dimensions, psychometric properties and validity.

We identified 20 original (G)EDI items eligible for measuring SR. Starting with these 20 items, we used exploratory factor analysis to assess constructs and dimensions using the development dataset. Cross-validation with both datasets using confirmatory factor analysis was successful and resulted in a 13-item, three-factor GEDI-SR scale model with excellent goodness of fit indices for measuring SR in kindergarten children. The GEDI-SR scale’s internal consistency, test–retest and interrater reliability, stability across populations as well as concurrent validity with the VSK-SR scale were in the good to excellent range, which qualifies the scale for screening or monitoring purposes. Since all items of this SR scale are inherent to the (G)EDI, SR can now be efficiently measured when administering the (G)EDI, without the need for applying an additional SR assessment instrument. Alternatively, given high reliability and validity, the newly developed, short GEDI-SR scale could also be administered as stand-alone scale.

### Development of the GEDI-SR scale and its constructs and dimensions

The sequence of theory-based selection process and a subsequent quantitative analysis of constructs and dimensions of the resulting eligible SR-items across two independent data sets was successful to reduce the initial 20 items to a very short scale of 13 items to measure SR in a valid way. The internal consistency of this scale was high (⍺ 〜 0.90).

The 13 items of the resulting SR scale revealed large correlations at the factor and item level, which indicates a multicomponent latent construct. The three factors of the GEDI-SR scale found empirically correspond perfectly to the theoretical basis of Diamond's conceptual model on SR [33], which underlines the scale’s validity. It consists of the “core” components of SR 1) behavioral response inhibition; 2) cognitive inhibition; 3) selective or focused attention (Diamond 2013). A child scoring high on these domains will find it easier to a) meet teachers' expectations, as teachers expect children to behave appropriately with regard to their school readiness and show SR by treating people and things well, by being able to sit still and to listen when needed [67]. Such children will show b) responsible behavior by following rules, taking responsibility for their actions, and being mindful of the materials and furniture at the kindergarten; c) concentration being able to conduct activities independently and calmly, e.g. completing painting and handicrafts carefully and on time, and to have an appropriate attention span. Children with high levels of SR may be expected to show d) conscientiousness, for example being careful with play materials.

The exploratory factor analysis led to omission of four items from the eligible SR-item selection. These encompass items such as “demonstrates self-control”, “has temper tantrums”, “has the ability to get along with peers” and “has difficulty awaiting turn in games or groups”, which -based on face-validity- might actually relate to the concept of SR. It is therefore not fully clear why the exploratory factor analysis suggested omission. The most probably hypothesis is that these items capture other behavioral domains distinct from the 13-items representing SR. Likewise, the structural equation modeling failed to support the inclusion of the items “gets into physical fights”, “is impulsive, acts without thinking” and “is able to follow class routines without reminders” – although all three investigators initially considered them to be appropriate and relevant items to measure SR. This however does not seem unusual: Also other studies on the development of theory- or literature-based questionnaires have shown that theoretically relevant items are dropped after factor analytic steps [68, 69]. Authors have argued that this might be due to the wording of some items not being appropriate to reflect the latent construct for which they were actually included.

### Reliability assessment

The 13-item GEDI-SR scale showed favorable reliability, both with respect to internal consistency as well as the results from structural equation modeling and re-test analyses. Yet, we must acknowledge some limitations regarding test–retest and interrater reliability. First, due to the COVID-19 pandemic and difficult organizational conditions in kindergartens, we received significantly fewer pairs of data than intended. With three pairs only for 6-year-olds, calculation of ICCs was not possible as was the calculation of interrater ICCs for 4- to 6-year-olds. We therefore only present overall values and recommend age-specific reliability analysis in a future study.

### Concurrent validity

We assessed concurrent validity by comparison to the VSK-SR scale. The VSK-SR scale tends to focus behavioral inhibition, namely patience, adaptability, and perseverance skills, whereas the GEDI-SR scale reflects cognitive inhibition and selective/focused attention with slightly different dimensions (concentration, diligence, and adherence to rules). Given this difference, the degree of agreement in terms of Pearson’s correlation coefficient was good. However, despite good overall concurrent validity results, the additional Bland–Altman analysis revealed that the two scales ((G)EDI-SR versus VSK-SR) differed for extreme values of SR. It thus remains uncertain whether the VSK-SR overestimates the extremes or the GEDI-SR underestimates deviations from the mean. Therefore, a future study might want to re-investigate the agreement of the GEDI-SR scale and another instrument available in German language, such as the SDQ.

### Comparison of reliability and validity results with those of other SR instruments

Regarding its psychometric properties and validity, the GEDI-SR scale shows values comparable (or even superior) to those of other instruments used to measure SR in the international and national context, as exemplified and quantified in Table 9. For example, the GEDI-SR scale compared to the other instruments shows very good internal consistency. Test–retest reliability seems even better than that of the CBQ or SDQ.

**Table 9:** Comparison of psychometric properties of the GEDI-SR scale with other SR-measurements

	**GEDI-SR**	**Other SR-Measurements**				
		CBCL 1,5–5	CBQ^a^	SDQ	BRIEF-P Canadian Sample	BRIEF-P German Sample
Psychometric properties	Current study	Achenbach (2000)	Putnam (2006)	Goodman (2001)	Shermand (2010)	Daseking (2013)
*Reliability*						
Internal consistency	⍺ 〜 0.90	⍺ ≥ 0.86	⍺ = 0.67 to 0.71	⍺ = 0.73	0.90 < ⍺ > 0.97	0.82 < ⍺ > 0.94
Test–retest reliability	ICC = 0.85 (95%-CI: 0.71 to 0.93)	Pearson’s *r* = 0.72 to 0.89	*r* = 0.61 – 0.70	*r* = 0.73 (after 4 to 6 months)	*r* = ≥ 0.90	X
Interrater reliability	ICC: 0.71 (95%-CI: 0.43 to 0.89)	*r* = 0.52 to 0.78	*r* = 0.47	*r* = 0.80; (sample of 5–15-Year-Olds)	X	*r* = 0.56
*Validity*						
Concurrent Validity	*r* = 0.75 with VSK-SR	*r* = 0.56 to 0.77 with the Richman Behavior Checklist	X	OR = 13.5 (95%-CI: 11.1 to 16.3) with DSM-IV-diagnosis	X	*r* = 0.70 with BASC^b^

Note: ^a^Values for parents as respondents; ^b^*BASC* Behavioral Assessment of Children (Reynolds & Kamphaus 2004), *X* Information not available, *ICC* Intraclass correlation coefficient

Moreover, our results confirm the good psychometric properties of the original (G)EDI and show that the "Social Competence" and "Emotional Maturity" scales of the EDI have been developed very well with regard to the selection and formulation of items. Building on this excellent work of the Canadian developers, we were now able to develop a reliable and valid SR scale that is inherent to the (G)EDI and thus does not require additional time for SR-assessment.

### Public health implication

Given good psychometric characteristics, high validity and reliability of the (G)EDI-SR scale, our work is the precondition for a public health monitoring process, which could take GEDI-SR as part of the (G)EDI or as a stand-alone scale as a starting point for intervention implementation, both at the individual child as well as the population level. The newly developed GEDI-SR might be specifically relevant to those countries already monitoring child development in kindergartens using the EDI at scale (e.g., Australia [45]). However, to lever its use as a potential public health screening instrument, in a next step, age-specific standardized cut-offs should be established in a representative sample (standardization sample) [70]. After the establishment of valid cut-off values, each country using the EDI for developmental monitoring could efficiently screen for SR difficulties in this early age and use the screening for tailored implementation of SR-promoting interventions in kindergartens at a public health scale.

### Strengths and limitations

To our best knowledge, this is the first study to define and validate a short SR scale within the widely used EDI. Although other short SR subscales exist (e.g. in the VSK-SR or the CBRS) and might be theoretically usable, our scale might be very efficient from a public health perspective as its items are part of and included in the administration of the EDI or GEDI. In addition, the costly purchase of e.g. the VSK (which is not open access) and the necessary, separate scoring methodology make the use of a separate SR scale potentially challenging for teachers and public health researchers, especially if compared to the (G)EDI assessment, which would allow developmental and SR assessment at once and is available free of charge.

In terms of item selection for the GEDI-SR scale, we only achieved a moderate agreement between raters, which underscores the difficulty to distinguish SR from other constructs such as social competence or emotional maturity. Despite the agreement and consensus regarding the theoretical basis, the only moderate agreement might also be explained by the raters’ different professional perspective and background (psychology, occupational therapy, pedagogy), e.g. bringing about different preferences for wordings and deviating operationalizations. However, reassuringly, the results of our exploratory and confirmatory factor analyses and structured equation modeling suggest that the selected items represent the latent construct SR.

Although we were able to include two independent data sets, we are aware that both might be affected by selection bias, according to their geographic location (e.g. potentially containing lower numbers of children from families with low socioeconomic status). As we did not collect the SES of the children's families we cannot assess representativeness of the samples. Hence, our data cannot readily be generalized to specific subgroups of interest, for example children from parents with recent migrant background and lower socio-economic or educational status. Moreover, 6-year-old children are underrepresented in both datasets. We found differing percentile values for lower age groups, but we attribute these to a higher inter- and intra-individual variability of developmental maturity [71].

In addition, we did not establish reference values in a representative data set. However, given the successful replication of the structured equation modeling with the validation dataset, we were at last able to demonstrate the stability of the model across populations. Last, at this stage and without a standardized sample, we are currently unable to determine the predictive validity of the GEDI-SR scale.

## Conclusion

Thirteen items in the (G)EDI can be recombined to a reliable and valid (G)EDI-SR scale, which can be used either as a stand-alone scale or as part of regular developmental monitoring using the EDI or GEDI in kindergartens. Through using the SR scale as part of (G)EDI kindergarten monitoring, kindergartens with higher percentages of children with SR difficulties could be identified and interventions implemented in a tailored way. Future research collecting data with the GEDI-SR in a representative sample could provide appropriate age- and domain-specific standardized cut-offs that would enable an adequate evaluation of area-wide population-based data.

## Data Availability

The datasets used and/or analyzed during the current study are available from the corresponding author on reasonable request.

## Abbreviations

BA: Bland-Altman
BIKO: Bildungskompetenzen Organisieren / BIKO-Screening zur Entwicklung von Basiskompetenzen für 3- bis 6-Jährige (Organizing Education in Preschool)
BRIEF-P: Behavior rating inventory of executive function—preschool version
CFI: Comparative fit index
CPD: Center for Preventive Medicine and Digital Health
DESK 3-6: Dortmunder Entwicklungs Screening für den Kindergarten (Dortmund Developmental Screening for Kindergarten)
GEDI: German version of the Early Development Instrument
GEDI-SR scale: GEDI self-regulation scale
ICC: Intraclass correlation coefficient
KOMPIK: Kompetenzen und Interessen von Kindern (Competencies and Interests of Children)
RMSEA: Root mean squared error of approximation
SDQ: Strengths and difficulties questionnaire
SES: Socioeconomic status
SR: Self-regulation
VSK: Verhaltenssalen für das Kindergartenalter (Kinergarten Behavior Scales)
VSK-SR: VSK self-regulation scale

## References

[1] Moffitt TE, Arseneault L, Belsky D (2011). A Gradient of Childhood Self-Control Predicts Health, Wealth, and Public Safety. Proc Natl Acad Sci.

[2] Robson DA, Allen MS, Howard SJ (2020). Self-regulation in childhood as a predictor of future outcomes: A meta-analytic review. Psychol Bull.

[3] Gawrilow C, Rauch W. Selbstregulationsfähigkeiten und exekutive Funktionen im Entwicklungsverlauf bei Vorschulkindern und Schulkindern. (Self-regulatory and executive functions over the developmental course in preschoolers and school-age children.). In: Hartmann U, Gold A, Marcus H (eds) Entwicklungsverläufe verstehen - Kinder mit Bildungsrisiken wirksam fördern - Forschungsergebnisse des Frankfurter IDeA-Zentrums. Stuttgart: Kohlhammer; 2017.

[4] Diamond A (2013). Executive functions. Annu Rev Psychol.

[5] Calkins SD, Graziano PA, Keane SP (2007). Cardiac vagal regulation differentiates among children at risk for behavior problems. Biol Psychol.

[6] Eisenberg N, Valiente C, Spinrad TL (2009). Longitudinal Relations of Children’s Effortful Control, Impulsivity, and Negative Emotionality to Their Externalizing, Internalizing, and Co-Occurring Behavior Problems. Dev Psychol.

[7] Nigg JT (2017). Annual Research Review: On the relations among self-regulation, self-control, executive functioning, effortful control, cognitive control, impulsivity, risk-taking, and inhibition for developmental psychopathology. J Child Psychol Psychiatry.

[8] Riggs NR, Kobayakawa Sakuma K-L, Pentz MA. Preventing Risk for Obesity by Promoting Self-Regulation and Decision-Making Skills. Pilot Results From the PATHWAYS to Health Program (PATHWAYS). Eval Rev. 2011;11:287–310. 10.1177/0193841X06297243 17478630

[9] Dierckens M, Richter M, Moor I, et al. Trends in material and non-material inequalities in adolescent health and health behaviours: A 12-year study in 23 European countries. Prev Med (Baltim); 157. Epub ahead of print 1 April 2022. 10.1016/j.ypmed.2022.107018. 10.1016/j.ypmed.2022.107018 35283161

[10] White BA, Jarrett MA, Ollendick TH (2013). Self-regulation deficits explain the link between reactive aggression and internalizing and externalizing behavior problems in children. J Psychopathol Behav Assess.

[11] Hölling H, Erhart M, Ravens-Sieberer U (2007). Verhaltensauffälligkeiten bei Kindern und Jugendlichen: Erste Ergebnisse aus dem Kinder- und Jugendgesundheitssurvey (KiGGS) (Behavioral problems in children and adolescents: Initial findings from the Child and Adolescent Health Survey.). Bundesgesundheitsblatt Gesundheitsforschung Gesundheitsschutz.

[12] Klipker K, Baumtarten F, Göbel K (2018). Mental Health Problems in Children and Adolescents in Germany. Results of the Cross-Sectional KiGGS Wave 2 Study and Trends. J Health Monit.

[13] Nakamura YM, Lehmann RJ (2003). Mitarbeiter/Innenbeurteilung. Lebens- und Schulqualität PH Akzente.

[14] Nodi M, Ackermann K, Eberhard U, et al. Arbeitsbedingungen, Belastungen und Ressourcen von Lehrpersonen und Schulleitungen im Kanton Aargau 2008. Ergebnisse der Untersuchung im Auftrag des Departements Bildung, Kultur und Sport. (Working Conditions, Burdens and Resources of Teachers and School Administrators in the Canton of Aargau 2008. Aarau: Results of the Study Commissioned by the Department of Education, Culture and Sport.); 2008.

[15] Keller R, Kunz A, Luder R, Zala-Mezö E, Strauss N-C, Häbig J (2018). Schulentwicklung für eine inklusive und gesunde Schule am Beispiel der Projekte „SIS“ und „Challenge“. (School development for an inclusive and healthy school using the example of the ‘SIS’ and ‘Challenge’ projects.). Dimensionen von Schulentwicklung. Verständnis, Veränderung und Vielfalt eines Phänomens. Münster.

[16] Campbell SB, Pierce EW, March CL (1994). Hard-to-Manage Preschool Boys : Symptomatic Behavior across Contexts and Time. Child Dev.

[17] Klora M, Zeidler J, Linder R (2015). Costs and treatment patterns of incident ADHD patients - a comparative analysis before and after the initial diagnosis -. Health Econ Rev.

[18] Ewest F, Reinhold T, Vloet TD (2013). Durch Jugendliche mit Störungen des Sozialverhaltens ausgelöste Krankenkassenausgaben: Eine gesundheitsökonomische Analyse von Versichertendaten einer gesetzlichen Krankenkasse. (Health insurance costs caused by adolescents with social behavior disorders: A Health Economic Analysis of Insured Data from a Public Health Insurance Fund.). Kindheit und Entwicklung.

[19] Blair C (2006). How similar are fluid cognition and general intelligence? A developmental neuroscience perspective on fluid cognition as an aspect of human cognitive ability. Behavioral and Brain Sciences.

[20] Ceci SJ (1991). How much does schooling influence general intelligence and its cognitive components? A reassessment of the evidence. Dev Psychol.

[21] Cicchetti D (2002). The impact of social experience on neurobiological systems: illustration from a constructivist view of child maltreatment. Cogn Dev.

[22] Diamond A, Lee K (2011). Interventions shown to aid executive function development in children 4 to 12 years old. Science..

[23] Pandey A, Hale D, Das S (2018). Effectiveness of universal self-regulation-based interventions in children and adolescents a systematic review and meta-analysis. JAMA Pediatr.

[24] Muir RA, Howard SJ, Kervin L (2023). Interventions and Approaches Targeting Early Self-Regulation or Executive Functioning in Preschools: A Systematic Review. Educ Psychol Rev.

[25] Blair C, Ku S. A Hierarchical Integrated Model of Self-Regulation. Front Psychol; 13. Epub ahead of print 4 March 2022. 10.3389/fpsyg.2022.725828. 10.3389/fpsyg.2022.725828 PMC8934409 35317011

[26] Epstein MH, Sharma JM (1998). Behavioral and Emotional Rating Scale: A strength-based approach to Assessment.

[27] Achenbach TM, Maruish ME (1999). The Child Behavior Checklist and related instruments. The use of psychological testing for treatment planning and outcomes assessment.

[28] Putnam SP, Rothbart MK (2006). Development of Short and Very Short Forms of the Children’s Behavior Questionnaire. J Pers Assess.

[29] Bronson MB, Goodson BD, Layzer JI (1990). Child behavior rating scale.

[30] Conners CK (1969). A teacher rating scale for use in drug studies with children. American J Psychiatry..

[31] Lebuffe PA, Naglieri JA (1998). The Devereux Early Childhood Assessment (for children ages 2 through 5 years).

[32] Lafreniere PJ, Dumas JE (1996). Social Competence and Behavior Evaluation in Children Ages 3 to 6 Years: The Short Form (SCBE-30). Psychol Assess..

[33] Gouley KK, Brotman LM, Huang KY (2008). Construct validation of the social competence scale in preschool-age children. Soc Dev.

[34] Goodman R (1997). The Strengths and Difficulties Questionnaire: a research note. J Child Psychol Psychiatry.

[35] Goodman R (2001). Psychometric Properties of the Strengths and Difficulties Questionnaire. J Am Acad Child Adolesc Psychiatry.

[36] Gioia GA, Isquith PK, Guy SC (2000). Behavior Rating Inventory of Executive Function. Child Neuropsychol.

[37] McCoy DC (2019). Measuring Young Children’s Executive Function and Self-Regulation in Classrooms and Other Real-World Settings. Clin Child Fam Psychol Rev.

[38] Daseking M, Petermann F. Verhaltensinventar zur Beurteilung exekutiver Funktionen für das Kindergartenalter Deutschsprachige Adaptation des Behavior Rating Inventory of Executive Function® - Preschool Version (BRIEF®-P) von Gerard A. Gioia, Kimberly Andrews Espy und Peter K. Isquith. Göttingen: Hogrefe; 2013.

[39] Koglin U, Petermann F (2016). Verhaltensskalen für das Kindergartenalter.

[40] Seeger D, Holodynski M, Souvignier E. Testbesprechung. BIKO-Screening zur Entwicklung von Basiskompetenzen für 3- bis 6-Jährige. (Test review. BIKO screening for the development of basic skills for 3- to 6-year-olds.). Hogrefe Publishing Group, 2014. Epub ahead of print January 2014. 10.1026/0049-8637/a000122.

[41] Tröster H, Flender J, Reineke D (2005). Dortmunder Entwicklungsscreening für den Kindergarten (DESK 3–6). (Dortmund Development Screening for Kindergarten.). Kindheit und Entwicklung.

[42] Bauer C, Krause M, Mayr T (2010). Kompetenzen und Interessen von Kindern. Beobachtungs- und Einschätzboten für Kinder von 3,5 bis 6 Jahre. (Children’s competencies and interests. Observation and assessment tools for children from 3.5 to 6 years.).

[43] Janus M, Offord DR (2007). Development and psychometric properties of the Early Development Instrument (EDI): A measure of children’s school readiness. Can J Behav Sci.

[44] Georg S, Bosle C, Fischer JE (2020). Psychometric properties and contextual appropriateness of the German version of the Early Development Instrument. BMC Pediatr.

[45] Brinkman SA, Gregory TA, Goldfeld S (2014). Data Resource Profile: The Australian Early Development Index (AEDI). Int J Epidemiol.

[46] Curtin M, Madden J, Staines A, et al. Determinants of vulnerability in early childhood development in Ireland: a cross-sectional study. BMJ Open; 3. Epub ahead of print 2013. 10.1136/bmjopen-2012-002387. 10.1136/bmjopen-2012-002387 PMC3657679 23674442

[47] Hagquist C, Hellström L (2013). The Psychometric Properties of the Early Development Instrument: A Rasch Analysis Based on Swedish Pilot Data. Soc Indic Res.

[48] Ip P, Li SL, Rao N (2013). Validation study of the Chinese Early Development Instrument (CEDI). BMC Pediatr.

[49] Woolfson LM, Geddes R, McNicol S (2013). A Cross-Sectional Pilot Study of the Scottish Early Development Instrument: A Tool for Addressing Inequality. BMC Public Health..

[50] Equity from the Start - The Early Development Instrument, https://edi.offordcentre.com/about/what-is-the-edi/ (accessed 14 December 2022).

[51] What is the EDI? (https://edi.offordcentre.com/about/what-is-the-edi/) [accessed 28th August 2023].

[52] Mukaka MM. Statistics Corner: A guide to appropriate use of Correlation coefficient in medical research, www.mmj.medcol.mw (2012). PMC3576830 23638278

[53] Bailey R, Jones SM (2019). An Integrated Model of Regulation for Applied Settings. Clin Child Fam Psychol Rev.

[54] Janus M (2006). The Early Development Instrument: A Tool for Monitoring Children’s Development and Readiness for School. Early Child Development: From Measurement to Action A Priority for Growth and Equity.

[55] Ludwig-Mayerhofer W. ILMES - Internet-Lexikon der Methoden der empirischen Sozialforschung. (ILMES - Internet Encyclopedia of Methods in Empirical Social Research.), http://wlm.userweb.mwn.de/Ilmes/ilm_f3.htm (2016, accessed 14 December 2022).

[56] Hu L, Bentler PM, Hu L. Cutoff criteria for fit indexes in covariance structure analysis : Conventional criteria versus new alternatives Cutoff Criteria for Fit Indexes in Covariance Structure Analysis : Conventional Criteria Versus New Alternatives. 5511. Epub ahead of print 2009. 10.1080/10705519909540118.

[57] Maccallum RC, Browne MW, Sugawara HM (1996). Power Analysis and Determination of Sample Size for Covariance Structure Modeling of fit involving a particular measure of model.

[58] Schumacker RE, Lomax RG (2015). A Beginner’s Guide to Structural Equation Modeling.

[59] Loehlin JC, Beaujean AA (2017). Latent Variable Models: An Introduction to Factor, Path, and Structural Equation Analysis.

[60] Kyriazos TA (2018). Applied Psychometrics: Sample Size and Sample Power Considerations in Factor Analysis (EFA, CFA) and SEM in General. Psychology.

[61] Koo TK, Li MY (2016). A Guideline of Selecting and Reporting Intraclass Correlation Coefficients for Reliability Research. J Chiropr Med.

[62] Altman DG, Bland JM (1983). Measurement in Medicine: The Analysis of Method Comparison Studies. The Statistician.

[63] Bland JM, Altman DG. Statistical methods for assessing agreement between two methods of clinical measurement. The Lancet. Epub ahead of print 1986. 10.1128/AAC.00483-18. 2868172

[64] Bennetts SK, Mensah FK, Westrupp EM, et al. The Agreement between Parent-Reported and Directly Measured Child Language and Parenting Behaviors. Front Psychol; 7. Epub ahead of print 2016. 10.3389/fpsyg.2016.01710. 10.3389/fpsyg.2016.01710 PMC5104739 27891102

[65] Bland MJ, Altman DG (2003). Applying the Right Statistics: Analyses of Measurement Studies. Ultrasound Obstet Gynecol.

[66] Cox NJ, Steichen TJ. CONCORD: Stata Module for Concordance Correlation. Statistical Software Components S404501, Boston College Department of Economics, https://ideas.repec.org/c/boc/bocode/s404501.html (2007, accessed 20 March 2020).

[67] Savina E (2021). Self-regulation in Preschool and Early Elementary Classrooms: Why It Is Important and How to Promote It. Early Childhood Educ J.

[68] Légare F, Borduas F, Freitas A, et al. Development of a Simple 12-Item Theory-Based Instrument to Assess the Impact of Continuing Professional Development on Clinical Behavioral Intentions. PLoS One; 9. Epub ahead of print 18 March 2014. 10.1371/journal.pone.0091013. 10.1371/journal.pone.0091013 PMC3958345 24643173

[69] Kumah EA, Bettany-Saltikov J, van Schaik P, et al. Development and validation of a questionnaire to assess evidence-based practice and evidence-informed practice knowledge, attitudes, understanding and behavior. Teaching and Learning in Nursing. Epub ahead of print 2023. 10.1016/j.teln.2023.07.006.

[70] Moosbrugger H, Kelava A. Testtheorie und Fragebogenkonstruktion. (Test theory and questionnaire development.). 2013. Epub ahead of print 2013. 10.1007/978-3-642-20072-4_2.

[71] Van Dijk M, Van Geert P, Diehl M, Hooker K, Sliwinski MJ (2016). The nature and meaning of intraindividual variability in development in the early life span. Handbook of intraindividual variablity across the life span.

